# Comparative Pharmacognosy, Chemical Profile and Antioxidant Activity of Extracts from *Phania matricarioides* (Spreng.) Griseb. Collected from Different Localities in Cuba

**DOI:** 10.3390/plants7040110

**Published:** 2018-12-14

**Authors:** Yamilet I. Gutiérrez, Ramón Scull, Lianet Monzote, Katia M. Rodríguez, Adonis Bello, William N. Setzer

**Affiliations:** 1 Department of Chemistry, Institute of Pharmacy and Food, Havana University, Coronela, Lisa, Havana 13600, Cuba; ygutierrez@infomed.sld.cu (Y.I.G.); rscull@ifal.uh.cu (R.S.); 2 Parasitology Department, Institute of Tropical Medicine “Pedro Kouri”, Havana 10400, Cuba; monzote@ipk.sld.cu; 3 Centro de Inmunología Molecular, Havana 10400, Cuba; katiam@cim.sld.cu; 4 Facultad de Ciencias Químicas, Universidad de Guayaquil, P.O. Box 0901-5738, Guayaquil 090514, Ecuador; adonisbello@gmail.com; 5 Department of Chemistry, University of Alabama in Huntsville, Huntsville, AL 35899, USA

**Keywords:** *Phania matricarioides*, phytochemical screening, HPLC profile, quercetin, FRAP, DPPH, plant extracts

## Abstract

*Phania matricarioides* (Spreng.) Griseb. is a traditionally used plant with various pharmacological properties. However, there are only scarce reports about the phytochemistry and biological activity of this plant. In this work, *P. matricarioides* was collected from three different localities of Cuba: PmB (collected in Bauta, Artemisa), PmC (collected in Cangrejeras, Artemisa), and PmI (collected in La Lisa, Havana), extracted with aqueous ethanol, and analyzed macroscopically and microscopically. The extracts were screened for phytochemical contents, analyzed by TLC and HPLC, and screened for antioxidant activity using the FRAP and DPPH assays. Macroscopic analysis showed similar results for all samples; however, microscopic, physicochemical and phytochemical studies showed appreciable differences. In particular, the total solid of PmC extract was higher (1.94 ± 0.03%) than the other samples. In HPLC profiles, quercetin was identified in the three samples and a greater similarity between samples PmB and PmI was observed. All samples demonstrated radical-scavenging antioxidant activity by the DPPH assay, which PmC also demonstrated the smaller (*p* < 0.05) value (IC_50_ = 27.4 ± 0.1 µg/mL), but was statistically superior (*p* < 0.05) to vitamin C (IC_50_ = 23.7 ± 0 µg/mL). Also, in the FRAP assay, a higher vitamin C equivalent of PmC was significantly superior (*p* < 0.05) to the other extracts at the evaluated concentrations, which is likely due to a higher concentration of quercetin. In conclusion, *P. matricarioides* could constitute a potential resource in the field of phytotherapeutic products, and the results obtained can contribute to the development of the quality control norms for this species.

## 1. Introduction

Asteraceae or Compositae is a very large and widespread family of flowering plants, with approximately 2000 genera [1]. Many members are grown as ornamental plants for their flowers, and are economically important, providing products such as cooking oils, lettuce, sunflower seeds, artichokes, sweetening agents, coffee substitutes and herbal teas [2]. In addition, many species possess medicinal properties, including antioxidant [3], antiparasitic [4], anticancer [5] and hepatoprotective [6].

**Figure 1:**
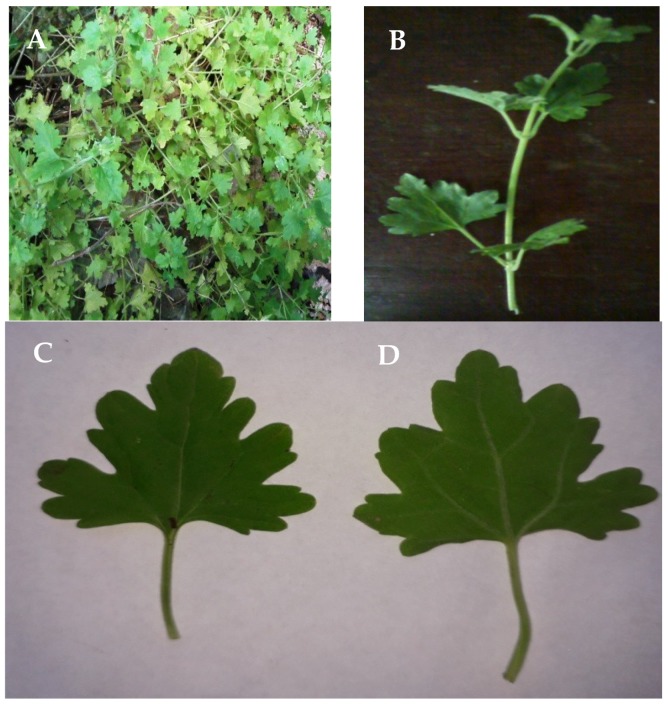
*Phania matricarioides*. (**A**): In the natural habitat; (**B**): sample in laboratory. (**C**): Top of the leaf; (**D**): back of the leaf. Photographs taken by the authors during and after the collection of the plant.

Among species of this family, *Phania matricarioides* (Spreng.) Griseb., the accepted name of *Hymenopappus matricarioides* Spreng, is a very branched plant that has white corollas (Figure 1). It is typical of stony and sandy places and has been reported in the Bahamas, Cuba, Curaçao, Jamaica, Margarita, Trinidad and Tobago and in Latin America from Mexico to Venezuela [7]. In general, scientific reports on the chemical composition and pharmacological effects of *P. matricarioides* are scarce. Nevertheless, related to therapeutic activity, *P. matricarioides* has been traditionally used for digestive conditions (stomach pain, bad digestion and diarrhea) and dermatological lesions [8,9].

In Cuba, the plant is also found throughout the island, cultivated in gardens and commonly known as chamomile, garden chamomile or chamomile of the earth [10]. As part of enhancing the knowledge about medicinal plants used by the Cuban people, Cabrera et al. [8] demonstrated the presence of tannins, flavonoids and other phenolic compounds, lactones, triterpenes or steroids, terpenes, and organic acids in phytochemical screening of extracts from *P. matricarioides*. In addition, the antidiarrheal, analgesic and anti-inflammatory effects have been demonstrated [11].

Herein, we have carried out a comparative analysis relating pharmacognosy, chemical profile and antioxidant activity of extracts from *P. matricarioides* collected in different localities of Cuba, which could be applicable in quality control assessment and therapeutic utility of this and other plants in the development of natural products.

## 2. Results

### 2.1. Macroscopic and Microscopic Analysis

**Figure 2:**
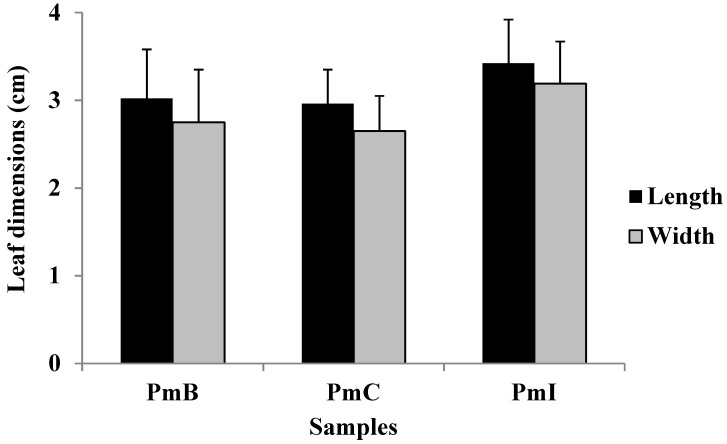
Leaf dimensions of *Phania matricarioides* collected from different geographical localities in Cuba. PmB: collected in Bauta, Artemisa Province, Cuba; PmC: collected in Cangrejeras, Artemisa Province, Cuba; PmI: collected in gardens of the Faculty of Pharmacy and Foods, La Lisa, Havana Province, Cuba. Results are the means with standard deviations of 100 leaves obtained from 20 plant samples from each area.

The leaves of three analyzed samples were pubescent, green color, closed venation (actinodroma), short petiole, oblong-oval contour, dentate–lobulate, rounded apex, truncated-subacorazonate base, sometimes obtuse or somewhat acute. Although the leaf dimensions of sample PhI appear to be higher, no statistical differences (*p* > 0.05) were found between the samples (Figure 2). The stems were green, cylindrical, herbaceous and pubescent.

**Figure 3:**
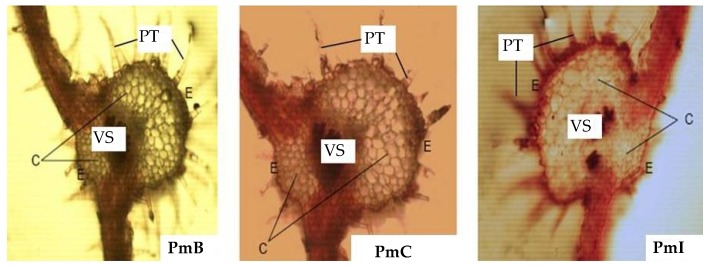
Photomicrograph of transverse sections of leaves from *Phania matricarioides* collected from different geographical localities in Cuba. (PmB): collected in Bauta, Artemisa Province, Cuba; (PmC): collected in Cangrejeras, Artemisa Province, Cuba; (PmI): collected in gardens of the Faculty of Pharmacy and Foods, La Lisa, Havana Province, Cuba; C: chlorenchyma; PT: pluricellular trichomes; VS: vascular system; E: epidermis. Photographs were taken by the authors with an optical microscope at 10× magnification.

In the microscopic study of the leaves (Figure 3), pluricellular trichomes (PT) were observed, which were more abundant in the PmB sample. The epidermis (E) was presented with a stratum of cells, but thicker in the PmI sample. The chlorenchyma showed spherical cells of different sizes, presenting larger in the histological section of PmI. The vascular system (VS) was compact in the samples of PmB and PmC, while disperse characteristics were observed for PmI.

**Figure 4:**
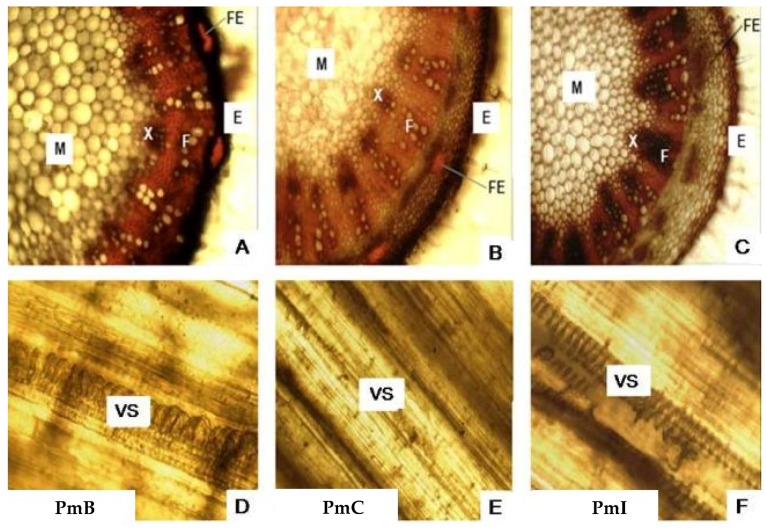
Photomicrograph of transverse and longitudinal sections of stems from *Phania matricarioides* collected from different geographical localities in Cuba. (**A**–**C**): transverse sections; (**D**–**F**): longitudinal sections. PmB: collected in Bauta, Artemisa Province, Cuba; PmC: collected in Cangrejeras, Artemisa Province, Cuba; PmI: collected in gardens of the Faculty of Pharmacy and Foods, La Lisa, Havana Province, Cuba; FE: sclerenchyma fibres; E: epidermis; X: xylem; F: phloem; M: pith; VS: vascular system. Photographs were taken by the authors with an optical microscope at 10× magnification.

Microscopic analysis of the stems (Figure 4A–C) allowed the visualization of the E with a layer of cells in the PmI samples, whereas in the PmB and PmC samples it has several layers of cells. Sclerenchyma fibers are present which are more compact in PmI sections. It was possible to observe xylem (X) and phloem (F) formed by well-defined cells, with a similar appearance in PmB and PmC. The pith (M) formed by spherical cells of variable size was observed in the center, but they were larger in the samples of PmB. The longitudinal sections of the stems (Figure 4D–F) showed a helical VS, which was larger in the longitudinal sections of PmB.

### 2.2. Physicochemical Analysis

The physicochemical parameters of *P. matricarioides* are listed in Table 1. No statistical differences (*p* > 0.05) were observed among samples collected in different geographical localities. However, appreciated differences (*p* < 0.05) in total solids were observed among extracts.

### 2.3. Phytochemical Screening

The content of total phenols and flavonoids showed statistical differences (*p* < 0.05) among samples (Table 1). In both cases, PmC displayed the higher content followed by PmB, while the smaller content was found in PmI.

A preliminary phytochemical analysis was conducted to identify the main chemical classes. In general, qualitative differences were not observed, characterized by a higher presence of phenols/tannins and absence of alkaloids, amino acids and saponins (Table 2). However, there were found differences in relative concentrations of the chemical classes. Coumarins and triterpenes/steroids were more evident in PmI and PmC, respectively; while reducing sugars appeared with higher concentrations in both PmI and PmC samples.

### 2.4. Qualitative Chemical Profile by Thin-Layer Chromatography (TLC)

**Figure 5:**
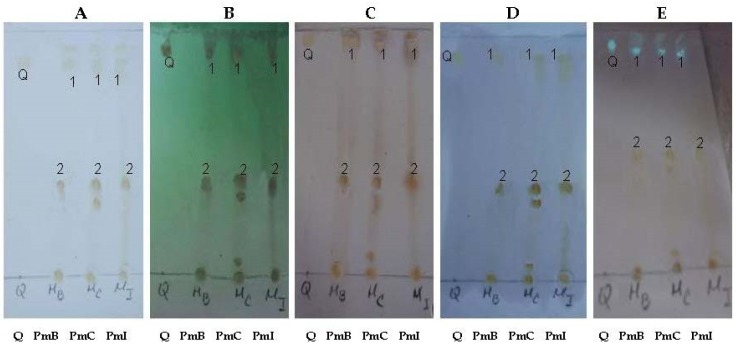
Chemical profile by TLC of extracts from *Phania matricarioides* collected from different geographical localities in Cuba. Quercetin was used as control. Aluminum-backed silica gel TLC plates. Mobile phase: *n*-butanol:acetic acid:water (65:25:10, *v*/*v*/*v*). (**A**): Visible; (**B**): UV 254 nm; (**C**): H_2_SO_4_/heat; (**D**): aluminum chloride 5%; (**E**): UV 365 nm; Q: quercetin; PmB: collected in Bauta, Artemisa Province, Cuba; PmC: collected in Cangrejeras, Artemisa Province, Cuba; PmI: collected in gardens of the Faculty of Pharmacy and Foods, La Lisa, Havana Province, Cuba.

Chemical components with different polarities were visualized by TLC. In this sense, phenolic compounds were observed in all samples, including the flavonoid quercetin, based on the retention factor (Rf) and color characteristics displayed (Figure 5). The PmC sample displayed the most complex chromatographic profile (Figure 5).

### 2.5. Analysis by High-Performance Liquid Chromatography (HPLC)

**Figure 6:**
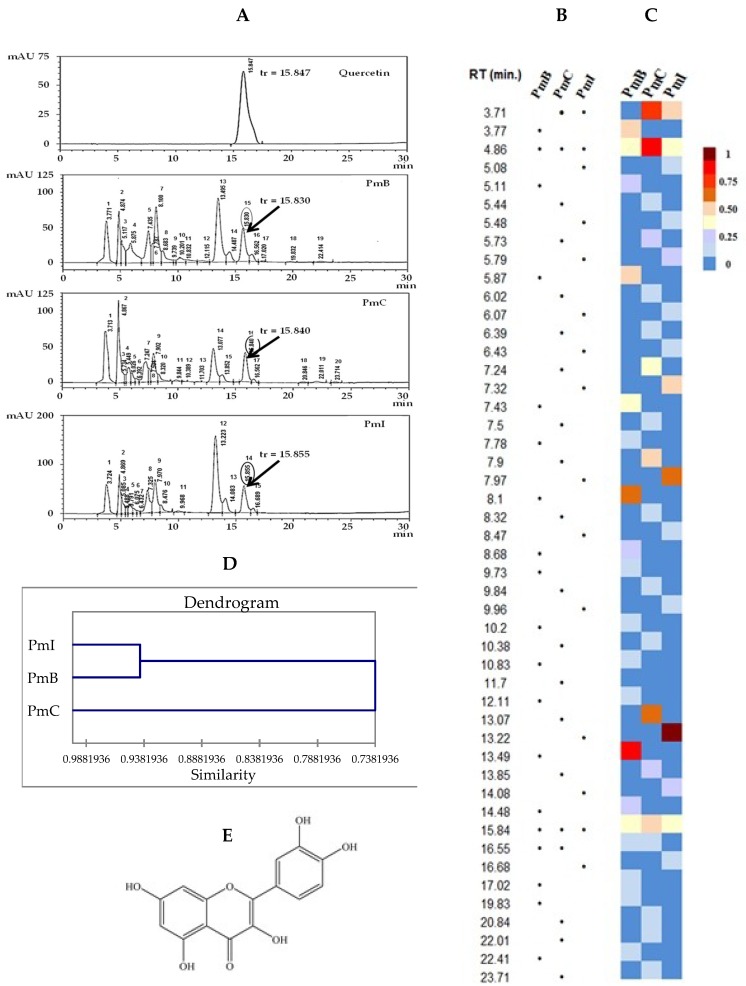
Chemical profile obtained by HPLC (Uptisphere 5 0DB 708965 RP-18 column, diode array detection) of extracts from *Phania matricarioides* collected from different geographical localities in Cuba. Quercetin was used as control. (**A**): Chromatograms (the arrows indicate the quercetin peak); (**B**): qualitative map visualization; (**C**): quantitative map visualization (the color range from red to blue indicates relative abundance from high to low); (**D**): dendrogram. (**E**): Chemical structure of quercetin. PmB: collected in Bauta, Artemisa Province, Cuba; PmC: collected in Cangrejeras, Artemisa Province, Cuba; PmI: collected in gardens of the Faculty of Pharmacy and Foods, La Lisa, Havana Province, Cuba; RT: retention time (min).

The profile obtained by HPLC showed qualitative and quantitative differences (Figure 6A). A total of 19, 20 and 15 peaks with a relative abundance according to the AUCs of 0.38–20.11%, 0.12–19.53% and 0.69–30.33% for PmB, PmC and PmI, respectively, was obtained. A general overview of the qualitative (Figure 6B) and quantitative (Figure 6C) compositions can also be visualized in the map. Among extracts, PmB and PmI resulted in closer chemical characteristics and constitute a cluster according to the obtained similarity dendrogram (Figure 6D). The results revealed that the flavonoid quercetin (Figure 6A,E) was identified in all the samples by comparison of its retention time and UV spectrum, although higher quantities were detected in the PmC extract (Figure 6C).

### 2.6. Antioxidant Activity

In this study, two different methods, ferric reducing antioxidant power (FRAP) and 2,2-diphenyl-1-picrylhydrazyl (DPPH) assays, were performed in order to quantify the antioxidant capacity and compare among three extracts. In the FRAP assay (Table 3), the vitamin C equivalents of PmC were significantly superior (*p* < 0.05) to other extracts at evaluated concentrations (25, 30 and 40 μg/mL). In addition, as summarized in Table 3, the PmC extracts exhibited percentages of DPPH radical sequestration statistically lower (*p* < 0.05) than the PmB and PmI, and higher (*p* < 0.05) than vitamin C.

## 3. Discussion

Medicinal plants have been used for centuries by humanity in the prevention and treatment of various diseases. However, the chemical constituents and biological properties of a wide variety of endemic plants used by people have been poorly studied and uncharacterized [12]. *Phania matricarioides* constitutes a species with medicinal properties and has been used in traditional herbal medicine. However, there is currently little information available about the plant, chemical composition remains unidentified and therapeutic value has not been scientifically demonstrated. From the above context, this study was designed to describe pharmacognostic characteristics, obtain the chemical profile and evaluate the antioxidant activity of three extracts from *P. matricarioides* collected from different localities in Cuba.

The macroscopic and microscopic characteristics of any plant to be used in the pharmaceutical industry should be considered as preliminary steps to establish their quality control profile. As per the guidelines of the World Health Organization (WHO) [13], pharmacognostic standards should be proposed as a protocol for the diagnosis and authentication of herbal drugs [14,15]. In the present study, the leaves from the PmI sample presented a larger size, possibly conditioned by the humidity and frequent drainage of the soil where the plant was developed. The macroscopic characteristics observed are in agreement with what has been reflected in the literature [7,10]. We have found no previous reports in the literature on the leaf size or macroscopic characteristics of the stems of this plant.

The microscopic analysis of the leaves and stems of the plants from the three collection sites displayed the possible structural differences that could accompany this plant as functions of the environmental conditions in which it is developed. In addition, accurate data on the climatological data of each region could also be a direct influence. The three zones or areas selected in general are subject to the same macroclimatic conditions; however, the conditions of habitat or ecological niche differ from one another, and are aspects that can cause some variations in the internal morphology of the plant.

The highest yield of soluble extractives was obtained for the 50% hydroalcoholic mixture, being higher in the PmC sample. In studies carried out by Cabrera et al., similar behavior was observed [8]. In the evaluations, a higher yield was obtained in extractions with hydroalcoholic mixture (22%); while extractions with ethanol and water extractives were smaller. Then, the main pharmacognostic parameters of the plant material were evaluated and corresponded to those reported in the literature for plant drugs [13,16,17], which will serve as a basis for establishing their quality limits. In this sense, it is known that changes of plant composition are probably due to geographic conditions, season to collection, which could impact the observed differences [18,19].

No differences were observed in the chemical composition determined by phytochemical screening in the evaluated samples. However, differences in the color intensity of some trials were observed, which could be related to different concentrations of functional groups. The results of the preliminary phytochemical screening are also in correspondence with those obtained by Cabrera et al. [8], demonstrating very clearly the presence of compounds of phenolic nature. Phenolics are important secondary metabolites in plants, of which several biological activities have been described [20,21].

Among the evaluated phytochemical parameters, differences in total solids were observed, which could bring relative information about quantities of metabolites or non-volatile constituents in the extracts. The PmC extracts displayed higher quantity of total solids, which could be in correspondence with the ecosystem of plant cultivation such as soil characteristics [19].

We then performed comparative analysis of the three extracts by HPLC, which is a versatile and very rapid tool that could be useful for chemotaxonomy, identification of the plant material, and to set up chemical fingerprints of studied extracts [22,23]. In addition, identification of phenolic compounds in medicinal plants is possible. The chromatograms displayed several compounds in the three extracts. Thus, the comparison of the chemical profiles of the extracts confirmed that the composition also depends on the geographical area, showing a wide profile of extracts from plants in the same country. Theses qualitative and quantitative differences were quickly assessed during the construction of related maps.

In the studied extracts, the presence of quercetin was confirmed, which reflects three important findings: (i) it could be a common compound present in *P. matricarioides* independent of locality of cultivation, (ii) it can be used as marker component in different collections, and (iii) it is a metabolite with known medicinal value [24,25,26,27], which suggests the need for further pharmacological screening of *P. matricarioides*-derived products. However, the metabolite maps of the extracts showed the accumulation patterns of quercetin to be different, also suggesting the need for further bioactivity screening. In addition, a peak with RT of 4.87 min was observed in the three studied samples. The UV spectrum is characteristic of chlorogenic acid or a derivative. This compound exhibited peaks at 325, 300 (shoulder) and 250 nm [28,29,30,31].

In recent years, natural antioxidants have gained importance for their prophylactic and therapeutic potential for many diseases, during which the search for medicinal plants with antioxidant properties has intensified in recent years [32,33]. Natural antioxidants are molecules that protect the organism from cellular damage resulting from excess free radicals responsible for inducing oxidative stress [33]. Oxidative stress is characterized by the imbalance between the production of oxidizing substances and endogenous antioxidants, and it may cause the oxidation of biomolecules such as nucleic acids, proteins and lipids [34]. In particular, phenolic compounds have been shown to be effective agents in scavenging reactive species responsible for inducing oxidative stress. In concordance with chemical studies, quercetin was identified in the three extracts, which has been noted in the literature for its antioxidant activities [35,36], and anticancer [37], neuroprotection [38], hepatoprotection [39], anti-inflammatory [40], and antiparasitic [41,42] properties. Among the *P. matricarioides* extracts, higher antioxidant activity was observed in the PmC extract, which also displayed the higher concentration of quercetin according to the chemical profile as shown in the quantitative map. Thus, it is likely that better antioxidant capacity may depend on quercetin concentration and locality collection area.

## 4. Materials and Methods

### 4.1. Plant Material

*Phania matricarioides* in the vegetative state was collected in March 2013, from three geographical localities: (i) Bauta (22°59′31″ N, 82°32′57″ W, 77 m asl), labeled as sample PmB, Artemisa Province, Cuba, which is characterized by a ferralitic soil, compact red clay, saturated in calcium, very plastic in the wet state and very hard or compact in the dry state; (ii) Cangrejeras (23°02′49″ N, 82°30′45″ W, 28 m asl), labeled as PmC, also from Artemisa Province and presents a typical red ferralitic soil, with high calcium content, good superficial and internal drainage; and (iii) gardens of the Faculty of Pharmacy and Food (IFAL) in La Lisa (23°01′29″ N, 82°27′47″ W, 60 m asl), labeled as PmI, Havana Province, Cuba, which is characterized by a red ferralitic soil, hydrated with humidity and frequent drainage. Plants were authenticated by Professor Dr. Jorge Gutiérrez Amaro and identified at the herbarium of National Botany Garden of Havana, Cuba, where the voucher specimen has been deposited under the number HFC 88669.

Vegetable material (leaves and stems) was washed with copious running water and immediately treated with 1% sodium hypochlorite solution for 5 min and then rinsed with copious running water. Fresh leaves and stems were used for macromorphology and micromorphology analyses. Another portion of sample (leaves and stems) was dried in a digital temperature controller VLD-6000 (AISET, Shanghai, China) with controlled temperature, at 35 °C ± 2 °C, over 7 days. The powdered sample (2 mm) was used for extract preparation, phytochemical analysis and phytochemical screening.

### 4.2. Preparation of Extracts

Hydroalcoholic extracts from vegetable material (leaves and stems) of *P. matricarioides* collected in the different localities belonging to the three collections were elaborated using a 50% hydroalcoholic mixture as solvent. Briefly, previously powdered sample was extracted by the maceration method, during a period of 7 days, at a temperature of 30 °C ± 2 °C according to the procedure described in the Cuban Standard Norm of Public Health (NRSP) 312 [43] and Martinez and Cuéllar [17].

### 4.3. Pharmacognostic Studies

To carry out the pharmacognostic studies, 20 samples of plant material (aerial parts) were collected from each geographical area. For macroscopic analysis, 100 leaves were included and size, shape, petiole, color, odor and extra features of leaves and stems from collected samples were studied [13,19]. For microscopic studies, free hand sections of leaves and stem were analyzed using optical microscopy (NOVEL, China) at 10× magnification, which were previously hydrated and rinsed with sodium hypochlorite, colored with safranin 1% and fixed with glycerine gelatin. In parallel, photomicrographs were taken with the digital camera HDCE-50B (Alltion (Wuzhou) Co. Ltd., Guangxi, China).

### 4.4. Physicochemical Analysis

The physicochemical analysis was carried out on the powdered sample (leaves and stems) following standard methods [13,19,44]. In this sense, moisture content (azeotropic distillation method), water-soluble extractive, alcohol-soluble extractive value at 50% and 90% (*v*/*v*) utilizing maceration method, total ash content, water-soluble ash and acid-insoluble ash were tested. Extracts were also characterized according to the Cuban standard NRSP 312 [43], including pH RS232 (Stark Instrument Company, Yantai, China), total solids, refractive index with an Abbe refractometer (Labmen Instrument Limited, Hong Kong, China), and relative density using a pycnometer. All tests were done in triplicate and results were expressed as mean and standard deviation (SD).

### 4.5. Phytochemical Analysis

Total contents of phenols and flavonoids were determined. In the first case, total phenol content was calculated determined by the Folin–Ciocalteu method [45], using gallic acid (Sigma-Aldrich, St. Louis, MO, USA) as the reference at concentrations of 10, 20, 30, 40 and 50 mg/mL. In addition, the total flavonoid content was carried out by the colorimetric method using aluminum chloride [46,47] and quercetin (Sigma-Aldrich) as the reference substance at the concentrations of 10, 15, 25, 50 and 100 μg/mL. In each case, a linear calibration curve was constructed with absorbance measured with a spectrophotometer Rayleigh UV-1601 (Beifen-Ruili Analytical Instrument Company, Beijing, China) at 715 nm. The total phenol and flavonoid contents (mg/mL, including standard deviations) were determined with respect to the reference calibration curves.

In addition, preliminary phytochemical screening for secondary metabolites was carried out. The powdered materials were extracted with solvents like petroleum ether, ethanol, and water. The extracts were tested for fats or oil, volatile oil, reducing sugars, resins, saponins, sterols, triterpenes, phenols/tannins, flavonoids, anthocyanins, free amino acids or amines, alkaloids, coumarins and quinones [19].

In order to corroborate qualitative chemical composition of the hydroalcoholic extracts of *P. matricarioides* from different localities, a profile by thin-layer chromatography (TLC) was also performed. Aluminum-backed silica gel TLC plates F 254 nm from Merck (Darmstadt, Germany) were used and quercetin (98% purity, from Sigma-Aldrich) was used as a standard. The chromatography was carried out using as mobile phase: *n*-butanol:acetic acid:water (65:25:10, *v*/*v*/*v*). The TLC plates were then sprayed with 5% H_2_SO_4_ in ethanol and heated to approximately 105 °C until the appearance of spots or modification of the appearance of existing ones. In another system, TLC was revealed with aluminum chloride (Merck, Germany) at 5% in methanol. Finally, TLC plates were visualized under UV light at 254/365 nm.

### 4.6. Analysis by High-Performance Liquid Chromatography (HPLC)

The samples were filtered through a Teflon membrane 0.2 µm and introduced directly into the equipment. A Shimadzu liquid chromatograph (Shimadzu, China) was used for the study. The chromatographic working conditions were: mobile phase acetonitrile and phosphate buffer pH 2.4 (70:30, *v/*v), oven temperature of 25 °C, detector by diode array at 370 nm (200–400), flow at 0.9 mL/min., pressure of 14.1 μPa, injection volume of 20 μL, column of Uptisphere 5 0DB 708965 (RP-18). Relative abundance was determined according to area under curve (AUC). Finally, chemical compositions of each sample were subjected to the heat map for qualitative and quantitative comparison. Finally, average of retention time (Rt) for peaks was performed and integrations were used to carry out the hierarchical clustering analysis using the XLSTAT software, version 2018.5.53172. Pearson correlation was selected as a measure of similarity, and the unweighted pair-group method with arithmetic average.

### 4.7. Antioxidant Activity

The ferric reducing antioxidant power (FRAP) assay was measured as described previously by Benzie and Strain [48], which determines the ability of the sample to reduce iron ferric (Fe^3+^) to ferrous (Fe^2+^). The determinations were carried out in a spectrophotometer Rayleigh UV-1601 (China) at 593 nm and concentration of FRAP was calculated from a linear equation obtained from a curve of vitamin C (purity 99%, Sigma-Aldrich) concentration vs absorbance. Different extract concentrations (ranging 25–40 μg/mL) were tested and equivalents μmol of vitamin C according to the standard curve of ascorbic acid (100, 200, 400, 800, 1000 μmol/L) were calculated. The experiment was carried out in triplicate and the results are expressed as vitamin C equivalents.

In parallel, the reduction of 2,2-diphenyl-1-picrylhydrazyl (DPPH; Sigma-Aldrich) radical to 2,2-diphenyl-1-picryl hydrazine was used for the radical-scavenging action of compounds containing –OH groups that decolorize the DPPH reagent [49]. Extracts were tested at different concentrations (ranging 25–50 μg/mL) and the absorbance was read at 517 nm in a spectrophotometer Rayleigh UV-1601 (China). The percentage inhibition of DPPH (% DPPH) staining was calculated by the following formula: % inhibition of the DPPH = (Abs control − Abs sample/Abs control) × 100. Results were expressed as mean and SD of medium inhibitory concentration (IC_50_), obtained from dose-response curves in Graphprism 5.0 Statistical Program of three replicates.

### 4.8. Statistical Analysis

To compare the macroscopic characteristics, physicochemical parameters and antioxidant activity among the samples of *P. matricarioides* collected in different localities, an analysis of variance was carried out, followed by the Dunnett or Tukey test. To antioxidant activity, data were analyzed by ANOVA test, followed by Duncan test. The analysis were carried out through the Statgraphics^®^Plus program, version 5.0, considering statistical differences as *p* < 0.05.

## 5. Conclusions

The pharmacognostic study performed for this plant species has established the micromorphologic features of leaves and stems, together with some physical–chemical parameters of plant material and extracts, which are essential for correct identification of plants and contribute to the development of the norms for quality control of the species. In addition, the identified compound (quercetin) constitutes a flavonoid with a broad spectrum of biological activities and could be used as a chemical marker of extracts. Finally, the antioxidant activity of these extracts could suggest pharmacological use of this plant and contribute to scientific validation of *P. matricarioides* as an herbal product, which has been used for centuries by the Cuban population.

In conclusion, *P. matricarioides* could constitute a potential resource in the field of phytotherapeutic products, expanding the chemical–pharmacognostic knowledge of the rich Cuban flora. Further experiments could investigate the antioxidant activity and other biological activities more deeply and examine this plant species for improvements of the pathologic status of patients with chronic diseases such as cancer and infections.

## Figures and Tables

**Table 1:** Physicochemical and phytochemical content of samples and extracts from *Phania matricarioides* collected from different geographical localities in Cuba.

Parameter	PmB	PmC	PmI
**Physicochemical Content ± SD in Vegetal Sample**			
Moisture content (%)	8.00 ± 0.00	8.73 ± 0.28	7.25 ± 0.35
Water-soluble extractive (%)	17.85 ± 0.06	14.76 ± 0.05	15.40 ± 0.09
Alcohol-soluble extractive at 50% (%)	18.37 ± 0.05	20.66 ± 0.02	17.60 ± 0.09
Alcohol-soluble extractive at 90% (%)	15.50 ± 0.06	13.22 ± 0.01	13.78 ± 0.02
Total ash content (%)	4.38 ± 0.15	3.77 ± 0.49	4.86 ± 0.14
Water-soluble ash (%)	2.98 ± 0.03	2.25 ± 0.03	2.69 ± 0.04
Acid-insoluble ash (%)	1.73 ± 0.06	1.24 ± 0.05	1.13 ± 0.01
**Physicochemical Content ± SD in Extracts**			
pH	5.72 ± 0.05	5.69 ± 0.10	5.80 ± 0.10
Total solid (%)	1.27 ± 0.01 ^a^	1.94 ± 0.03 ^b^	1.09 ± 0.06 ^c^
Refraction index	1.36 ± 0.00	1.35 ± 0.00	1.36 ± 0.00
Relative density (g/mL)	0.85 ± 0.00	0.86 ± 0.00	0.86 ± 0.00
**Phytochemical Content ± SD in Extracts**			
Total phenol (mg/mL)	32.57 ± 0.45 ^a^	41.38 ± 0.19 ^b^	24.75 ± 0.57 ^c^
Total flavonoid (mg/mL)	25.48 ± 0.14 ^a^	33.17± 0.04 ^b^	18.63 ± 0.24 ^c^

SD: standard deviation. PmB: collected in Bauta, Artemisa Province, Cuba. PmC: collected in Cangrejeras, Artemisa Province, Cuba. PmI: collected in gardens of the Faculty of Pharmacy and Foods, La Lisa, Havana Province, Cuba. Letters a, b, and c represent statistical differences (*p* < 0.05) among the studied extracts.

**Table 2:** Phytochemical analysis of samples (leaves and stems) from *Phania matricarioides* collected from different geographical localities in Cuba.

Test for Constituent Groups	Name of Test	Samples		
		PmB	PmC	PmI
Alkaloids	Dragendorff reagent test	−	−	−
Amino acid	Ninhydrin	−	−	−
Anthocyanins	HCl conc./pentanol	+	+	+
Coumarins	Baljet test	+	+	++
Flavonoids	Shinoda (Mg-HCl)	+	+	+
Phenols/tannin	Ferric chloride test	++	++	++
Quinones	Börntrager	+	+	+
Saponins	Foam test	−	−	−
Triterpens/steroids	Lieberman Burchard reagent	+	++	+
Reductant sugars	Fehling test	+	++	++
Fats or oil	Sudan III	+	+	+
Volatile oil	Microsublimation-vanillic	+	+	+

PmB: collected in Bauta, Artemisa Province, Cuba. PmC: collected in Cangrejeras, Artemisa Province, Cuba. PmI: collected in gardens of the Faculty of Pharmacy and Foods, La Lisa, Havana Province, Cuba. −: Negative, +: positive, ++: highly positive.

**Table 3:** Antioxidant activity of different extracts from *Phania matricarioides* collected from different geographical localities from Cuba.

Products	FRAP Equivalents of Vitamin C (μM)			DPPH IC_50_ ± SD (μg/mL)
	25 μg/mL	30 μg/mL	40 μg/mL	
PmB	245.8 ±19.4 ^a^	366.5 ± 15.9 ^c^	768.1 ± 21.2 ^e^	28.3 ± 0.1 ^h^
PmC	344.3 ± 14.9 ^b^	525.2 ± 16.7 ^d^	869.7 ± 15.9 ^f^	27.4 ± 0.1 ^i^
PmI	249.0 ± 20.6 ^a^	357.0 ± 22.6 ^c^	726.0 ± 12.0 ^g^	28.6 ± 0.1 ^h^
Vitamin C	-	-	-	23.7 ± 0 ^j^

PmB: collected in Bauta, Artemisa Province, Cuba. PmC: collected in Cangrejeras, Artemisa Province, Cuba. PmI: collected in gardens of the Faculty of Pharmacy and Foods, La Lisa, Havana Province, Cuba. FRAP: ferric reducing antioxidant power assay. DPPH: assay with 2,2-diphenyl-1-picrylhydrazyl. Letters a, b, c, d, e, f, g, h, I and j represent statistical differences (*p* < 0.05) among studied extracts.
